# Analysis of expressed sequence tags generated from full-length enriched cDNA libraries of melon

**DOI:** 10.1186/1471-2164-12-252

**Published:** 2011-05-20

**Authors:** Christian Clepet, Tarek Joobeur, Yi Zheng, Delphine Jublot, Mingyun Huang, Veronica Truniger, Adnane Boualem, Maria Elena Hernandez-Gonzalez, Ramon Dolcet-Sanjuan, Vitaly Portnoy, Albert Mascarell-Creus, Ana I Caño-Delgado, Nurit Katzir, Abdelhafid Bendahmane, James J Giovannoni, Miguel A Aranda, Jordi Garcia-Mas, Zhangjun Fei

**Affiliations:** 1 URGV Plant Genomics, Unité de Recherche en Génomique Végétale, UMR1165 ERL8196 INRA-UEVE-CNRS. 2, Rue Gaston Crémieux, 91057 Evry, France; 2 Molecular and Cellular Imaging Center, The Ohio State University, OARDC, 1680 Madison Ave, Wooster, OH 44691, USA; 3 Boyce Thompson Institute, Cornell University, Ithaca, NY 14853, USA; 4 Centro de Edafología y Biología Aplicada del Segura (CEBAS), Consejo Superior de Investigaciones Científicas (CSIC), Apdo. Correos 164, 30100 Espinardo, Murcia, Spain; 5 IRTA, Center for Research in Agricultural Genomics CSIC-IRTA-UAB, Campus UAB, Edifici CRAG, 08193 Bellaterra (Barcelona), Spain; 6 Department of Vegetable Research, Agricultural Research Organization, Newe Ya'ar Research Center, P.O. Box 1021, Ramat Yishay, 30095, Israel; 7 Department de Genètica Molecular, Center for Research in Agricultural Genomics CSIC-IRTA-UAB, Campus UAB, Edifici CRAG, 08193 Bellaterra (Barcelona), Spain; 8 Department of Plant Production, College of Food and Agricultural Sciences, King Saud University, Riyadh Saudi Arabia; 9 USDA Robert W. Holley Center for Agriculture and Health, Tower Road, Ithaca, NY 14853, USA; 10 Seminis Vegetable Seeds, 37437 State Highway 16 Woodland, CA 95695, USA

## Abstract

**Background:**

Melon (*Cucumis melo*), an economically important vegetable crop, belongs to the *Cucurbitaceae *family which includes several other important crops such as watermelon, cucumber, and pumpkin. It has served as a model system for sex determination and vascular biology studies. However, genomic resources currently available for melon are limited.

**Result:**

We constructed eleven full-length enriched and four standard cDNA libraries from fruits, flowers, leaves, roots, cotyledons, and calluses of four different melon genotypes, and generated 71,577 and 22,179 ESTs from full-length enriched and standard cDNA libraries, respectively. These ESTs, together with ~35,000 ESTs available in public domains, were assembled into 24,444 unigenes, which were extensively annotated by comparing their sequences to different protein and functional domain databases, assigning them Gene Ontology (GO) terms, and mapping them onto metabolic pathways. Comparative analysis of melon unigenes and other plant genomes revealed that 75% to 85% of melon unigenes had homologs in other dicot plants, while approximately 70% had homologs in monocot plants. The analysis also identified 6,972 gene families that were conserved across dicot and monocot plants, and 181, 1,192, and 220 gene families specific to fleshy fruit-bearing plants, the *Cucurbitaceae *family, and melon, respectively. Digital expression analysis identified a total of 175 tissue-specific genes, which provides a valuable gene sequence resource for future genomics and functional studies. Furthermore, we identified 4,068 simple sequence repeats (SSRs) and 3,073 single nucleotide polymorphisms (SNPs) in the melon EST collection. Finally, we obtained a total of 1,382 melon full-length transcripts through the analysis of full-length enriched cDNA clones that were sequenced from both ends. Analysis of these full-length transcripts indicated that sizes of melon 5' and 3' UTRs were similar to those of tomato, but longer than many other dicot plants. Codon usages of melon full-length transcripts were largely similar to those of Arabidopsis coding sequences.

**Conclusion:**

The collection of melon ESTs generated from full-length enriched and standard cDNA libraries is expected to play significant roles in annotating the melon genome. The ESTs and associated analysis results will be useful resources for gene discovery, functional analysis, marker-assisted breeding of melon and closely related species, comparative genomic studies and for gaining insights into gene expression patterns.

## Background

Melon (*Cucumis melo*) belongs to the *Cucurbitaceae *family, which comprises 130 genera, including approximately 800 species that are mainly found in temperate, subtropical and tropical regions worldwide [1,2]. Besides melon, the *Cucurbitaceae *family also consists of many other economically important species, including cucumber (*C. sativus*), watermelon (*Citrullus lanatus*), squash and pumpkin (*Cucurbita *spp.). Economically, melon is among the most important fleshy fruits for fresh consumption. Indeed, melon is one of America's, Europe's and the Middle East's favorite fruits for dessert and salad uses because of its unique flavor. The average *per capita *consumption of melon in the U.S. has been increasing consecutively each decade since the 1960s with 2000-2006 average *per capita *consumption exceeding 12 pounds per year, an 8% rise from 1990-1999. Besides its economic importance, melon is a very useful experimental system for fundamental studies on a range of topics including sex determination [3,4] and vascular biology [5,6]. In addition, melon is also an intensively studied species in terms of fruit ripening. It exhibits extreme diversity for fruit traits and includes a wide variety of cultivars producing fruits differing in many traits including fruit shape, size, flesh color, sweetness, aroma volatiles and fruit texture [7]. In addition, melon fruits also have significant variations in ripening physiology and can be categorized as either climacteric or non-climacteric types based on their ripening related respiration rate and ethylene evolution profiles [8]. Extensive molecular and genetic studies have been carried out in recent years in order to better understand the regulatory mechanisms underlying important traits of melon with the aim to improve melon fruit quality [9,10]

Melon is a diploid species (2n = 24) with an estimated genome size of 450 Mb [11]. Genetic and genomic tools available in melon include BAC libraries [12-14], a physical map [15], high-resolution genetic maps [16-19], oligo-based microarrays [20], and a TILLING platform for functional studies [21]. Currently the melon genome is being sequenced under the Spanish Genomics Initiative (MELONOMICS) and the genome sequencing should be completed in the near future. The sequence of the closely related cucumber genome is available [22]. Complementary to whole genome sequences, expressed sequence tags (ESTs) can directly represent the transcriptome or transcribed portions of the genome. They have played significant roles in rapid gene discovery, improving genome annotation, elucidating phylogenetic relationships, facilitating breeding programs, and large-scale expression analysis [23]. Currently in the NCBI dbEST database, there are approximately 35,000 melon ESTs, most of which were produced by González-Ibéas et al. [24]. Approximately 8,000 ESTs are available for cucumber and watermelon, respectively, and a total of approximately 1,000 EST from other cucurbit species. Recently several reports have described the generation of large-scale transcriptome sequences in cucurbit species using next generation sequencing technologies (mainly the Roche-454 massive parallel pyrosequencing technology), including melon [25], cucumber [26], and *Cucurbita pepo *[27]. Although sequences generated under these efforts are much shorter than traditional Sanger ESTs, they represent a significant expansion of cucurbit functional genomics resources.

We undertook to expand the melon transcript catalog in the framework of the International Cucurbit Genome Initiative, which was established in 2005, being one of its major objectives to sequence approximately 100,000 ESTs from different melon genotypes and tissues [28]. We have constructed eleven full-length enriched cDNA libraries and four standard cDNA libraries from various melon tissues and cultivars and generated ~94,000 ESTs. These melon ESTs were analyzed to determine the structure and putative functions of the corresponding transcripts. In addition, a number of new SSR and SNP markers were identified in this EST collection. All of this data has been integrated in the Cucurbit Genomics Database [28]. The ESTs generated from the present study, especially those from full-length enriched cDNA libraries, will be a useful resource for the ongoing melon whole genome sequencing project and for characterizing gene expression patterns and traits of interest in melon and closely related species.

## Results and discussion

### Construction and sequencing of melon cDNA libraries

We constructed eleven full-length enriched and four standard cDNA libraries from various melon tissues (cotyledon, leaf, root, flower, fruit and callus) and cultivars (Dulce, PI161375, Piel de Sapo T-111, and Vedrantais) under normal conditions or upon infection with melon necrotic spot virus (MNSV)-Mα5 (Table 1). The flower, fruit and callus libraries were derived from two climacteric (Dulce and Vedrantais) and two non-climacteric cultivars (Piel de sapo T-111 and PI161375). For the flower and fruit, RNA pools were prepared from various developmental stages (see Methods). The leaf, root and cotyledon libraries were constructed from tissues infected with MNSV-Mα5. EST sequencing was carried out independently on full-length enriched and standard cDNA clones. For full-length enriched cDNA libraries, 70,576 randomly-selected clones were sequenced from the 5' end, producing 69,196 (98%) useful reads after trimming vector, adaptor and low-quality sequences and identifying and removing all possible contaminated sequences. Assembly of these ESTs produced 6,469 clusters, among which 2,721 non-redundant clones were selected for 3' end sequencing, yielding a total of 2,381 (87.5%) high quality 3' reads. For the four standard callus libraries, 26,112 randomly-selected clones were sequenced from the 5' end, generating 22,179 (85%) high quality EST sequences. In total, we have generated 93,756 high quality melon ESTs from the constructed cDNA libraries (Table 1) and the average length of these ESTs is 629.6 bp. The EST sequences have been deposited in GenBank and are also available at the Cucurbit Genomics Database [28].

**Table 1:** Description of melon cDNA libraries and summary of melon ESTs

Library	Cultivar	Tissue	No. 5' sequences	No. 3' sequences	Total sequences
**Full-length cDNA library**					
PFTP2	PI161375	mixture of fruits in four developmental stages	12,673	547	13,220
SFTP2	Piel de Sapo T-111	mixture of fruits in four developmental stages	3,630	139	3,769
VFTP2	Vedrantais	mixture of fruits in four developmental stages	3,621	149	3,770
DFTP2	Dulce	mixture of fruits in four developmental stages	3,549	70	3,619
VFLP2	Vedrantais	mixture of flowers in three developmental stages	19,261	638	19,899
PFLP2	PI161375	mixture of flowers in three developmental stages	3,548	62	3,610
DFLP2	Dulce	mixture of flowers in three developmental stages	3,411	126	3,537
SFLP2	Piel de Sapo T-111	mixture of flowers in three developmental stages	320	0	320
MNFG2	Piel de Sapo T-111	leaf infected by melon necrotic spot virus (MNSV)	7,776	295	8,071
MNRP2	Piel de Sapo T-111	root infected by melon necrotic spot virus (MNSV)	7,953	297	8,250
MNCP2	Piel de Sapo T-111	cotyledon infected by melon necrotic spot virus (MNSV)	3,454	58	3,512
**Subtotal No. sequences**			69,196	2,381	71,577
**Standard cDNA libraries**					
CM-DEa	Dulce	callus	5,485	0	5,485
CM-PEa	PI161375	callus	5,527	0	5,527
CM-TEa	Piel de Sapo	callus	5,700	0	5,700
CM-VEa	Vedrantais	callus	5,467	0	5,467
**Subtotal No. sequences**			22,179	0	22,179
**Total**			91,375	2,381	93,756

### Melon EST sequence assembly and annotation

**Figure 1:**
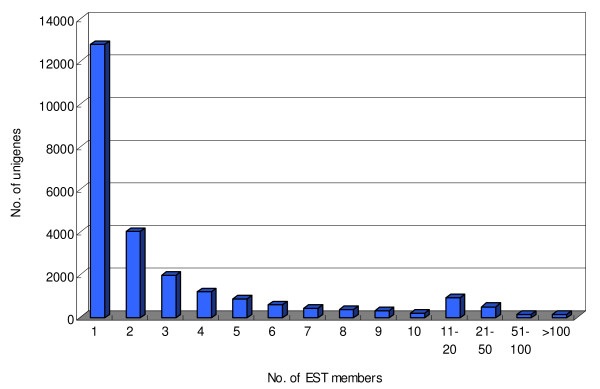
**Histogram of number of ESTs in each melon unigene**.

The 93,756 high quality melon ESTs generated under this study, together with ~35,000 ESTs that are publicly available [24,28,29] and 173 published mRNA sequences, were assembled into a melon unigene build. The resulting assembly contained a total of 24,444 unigenes with an average length of 776.7 bp, among which 11,653 were contigs with an average length of 972 bp and 12,791 were singletons with an average length of 598.7 bp (Table 2). The distribution of the number of ESTs in each melon unigene is shown in Figure 1. A number of highly abundant genes could be identified, with 162 unigenes represented by over 100 ESTs. The most abundant genes in the combined set of libraries (> 500 ESTs) are listed in Table 3. Details of the melon EST assembly are available at the Cucurbit Genomics Database [28].

**Table 2:** Statistics of melon unigenes

	Singleton	Contig	Unigene
**No. of sequences**	12,791	11,653	24,444
**Average read length (bp)**	598.7	972.0	776.7
**Total bases (bp)**	7,658,604	11,326,166	18,984,770

**Table 3:** Most abundant melon unigenes (>500 EST members)

Unigene ID	No. of ESTs	GenBank nr hit description	E value
MU46026	2054	ELP (EXTENSIN-LIKE PROTEIN); lipid binding	1e-31
MU45978	1120	type I proteinase inhibitor-like protein	9e-06
MU46015	915	acyl carrier protein	1e-25
MU45913	827	type-2 metallothionein	2e-19
MU45877	819	No hits found	
MU45416	786	No hits found	
MU45994	785	B12D-like protein	3e-38
MU46019	683	Wound-induced proteinase inhibitor 1	1e-10
MU45854	626	No hits found	
MU45964	619	lipid binding protein	3e-16
MU43757	618	60s acidic ribosomal protein	4e-23
MU47776	594	histone cluster 2, H3c2-like	2e-60
MU45763	591	type-2 metallothionein	2e-19
MU47828	580	chloroplast photosystem II 10 kDa protein	1e-52
MU45654	568	chlorophyll A/B binding protein	7e-147
MU45963	554	histone H4	3e-38
MU45282	514	ascorbate peroxidase	1e-120
MU45991	509	ubiquitin carrier-like protein	4e-83

Putative functions of melon unigenes were accessed by comparing unigene sequences against the GenBank non-redundant (nr) protein database using the NCBI BLAST program. The analysis showed that applying an e value cutoff of 1e-5, a total of 19,359 (79.2%) melon unigenes had hits in the nr database; while a total of 10,068 (41.2%) had hits when an e value cutoff of 1e-50 was applied. This indicated that a very high percentage of melon unigenes could be assigned a putative function. Those having no hits in the database are likely to include non-coding RNAs, genes whose sequences do not capture regions that contain conserved functional domains, or protein coding genes that are novel in the database and/or are melon-specific.

We then further compared melon unigenes to the pfam protein domain database [30]. A total of 8,251 (33.8%) melon unigenes contained at least one pfam domain and a total of 2,206 distinct pfam domains were represented by these 8,251 melon unigenes. A similar analysis on the well-annotated Arabidopsis proteins (TAIR version 10) indicated that 3,272 pfam domains could be represented by the Arabidopsis proteome. This suggested that melon unigenes assembled in the present study captured a large portion (at least 70%) of genes in the melon genome. The most highly represented pfam domains in the melon unigene database included PF00069 (protein kinase; 144 unigenes), PF00076 (RNA recognition motif; 138 unigenes), PF07714 (protein tyrosine kinase; 108 unigenes) and PF00097 (Zinc finger, C3HC4 type; 103 unigenes).

Based on BLAST and pfam annotations, melon unigenes were further annotated with Gene Ontology (GO) terms. A total of 15,350 (62.8%) unigenes were assigned at least one GO term, among which 12,953 (53%) were assigned at least one GO term in the biological process category, 13,149 (53.8%) in the molecular function category and 12,420 (50.8%) in the cellular component category; while 9,927 (40.6%) melon unigenes were annotated with GO terms from all the three categories. Based on the GO annotations, putative gene functions of melon unigenes were classified into high-level plant specific GO slims [31] in each of the three categories. The most abundant GO slims within the biological process, molecular function, and cellular component categories were cellular process, binding, and membrane, respectively. In addition, a large number of melon unigenes appeared to be involved in plant responses to abiotic (1,534) and biotic (844) stimuli, flower development (347), and secondary metabolite process (603), or have transcription factor activities (519).

To gain insights into metabolism-related genes, we further predicted biochemical pathways from the melon unigenes and built a melon metabolic pathway database using the Pathway Tools software [32]. A total of 302 metabolic pathways, as well as 30 superpathways, were predicted from 3,543 enzyme-coding melon unigenes. Most primary and secondary metabolic pathways were well-represented by melon unigenes. The melon metabolic pathway database is freely available at the Cucurbit Genomics Database [28].

### Quality assessment of melon full-length enriched cDNAs

As shown in Table 1, a total of 71,577 ESTs derived from full-length enriched cDNA clones were obtained in the present study. These ESTs were assembled into 6,848 unigenes, among which 6,469 contained 5' sequences of at least one full-length enriched cDNA clone. By blasting sequences of the 6,469 unigenes against GenBank nr, SwissProt/TrEMBL and Arabidopsis (TAIR version 10) protein databases, 5,552 (85.8%) had significant hits (1e-05). Out of the 5,552 unigenes, 4,668 (84.1%) hit within five amino acids of the corresponding start sites. This indicated that a large portion of clones from full-length enriched cDNA libraries encoded full-length cDNAs.

**Figure 2:**
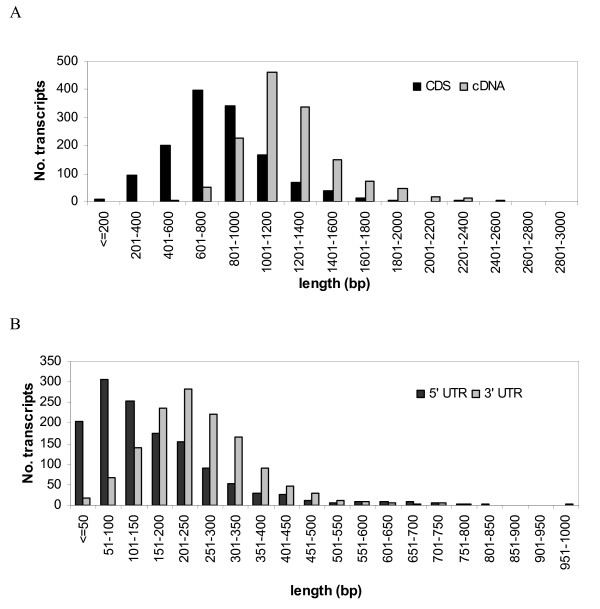
**Size distribution of cDNAs, CDS (A) and 5' and 3' UTRs (B) of melon full-length transcripts**.

We further generated 3' end sequences of more than 2,300 clones (Table 1) and ultimately obtained 2,162 clones that were sequenced from both the 5' and 3' ends, among which 1,538 (72.5%) had 5' and 3' sequences that were assembled into the same unigene. After removing redundancy, a total of 1,382 unigenes that contained 5' and 3' sequences of at least one full-length enriched cDNA clone were identified as melon full-length transcripts. The majority of the identified full-length transcripts contained overlapping 5' and 3' sequences from the same clone. The length distribution of melon full-length transcripts is shown in Figure 2A. The full-length transcripts ranged from 269 to 2,839 bp and their average size was 1,230 bp, which was shorter than previously reported for tomato (1,418 bp; [33]), Arabidopsis (1,445 bp; [34]), and soybean (1,539 bp; [35]), but longer than poplar (1,045 bp; [36]). We then predicted the complete protein-coding sequences (CDS) for the 1,382 melon full-length transcripts and were able to obtain CDS for 1,345 (97.3%) full-length transcripts. The remaining 37 could be non-coding RNAs or transcripts that did not contain full CDS. Indeed, we found that four transcripts (e.g., MU51348) did not contain a stop site. The average length of the predicted CDS was 814 bp, which was shorter than that of tomato (938 bp; [33]) and soybean (1,042 bp; [35]), but longer than poplar (649 bp; [36]) and maize (799 bp; [37]). The size distribution of melon CDS predicted from melon full-length transcripts is illustrated in Figure 2A. Overall, the average lengths of both melon full-length transcripts and CDS were shorter than those reported for full-length cDNAs of other plant species such as tomato [33], Arabidopsis [34], and soybean [35]. This is not unexpected since, as mentioned earlier, the majority of melon full-length transcripts were identified based on the overlap between 5' and 3' sequences of a single full-length cDNA clone.

Based on the predicted CDS, we extracted 5' and 3' UTR sequences for each melon full-length transcript. The average lengths of melon 5' and 3' UTRs were 167 bp and 254 bp, respectively, which were very close to those of tomato (175 bp and 257 bp, respectively) and longer than those of other plant species except rice [33]. The length distributions of melon 5' and 3' UTRs are shown in Figure 2B, which were also largely similar to those of tomato [33].

We further examined codon usages of the 1,345 melon full-length transcripts and compared the codon usages to those of Arabidopsis coding sequences (TAIR version 10). The statistics of the complete codon usages of melon and Arabidopsis CDS are provided in Additional file 1. Overall codon usages of melon full-length transcripts were largely similar to those of Arabidopsis CDS. TGA, TAA, and TAG accounted for 44.9%, 37.2%, and 17.9%, respectively, of melon stop codons; and they accounted for 43.6%, 36%, and 20.4%, respectively, of Arabidopsis stop codons (Additional file 1). In addition, the GC content of melon coding sequences (45.61%) was also very close to that of Arabidopsis (44.14%). This, combined with the evidence described above, supported the high quality of melon full-length enriched cDNA libraries.

### Comparative genomics analysis with other plants

To date, genome sequences of fourteen plant species have been published. These plant species are Arabidopsis [38], rice [39], poplar [40], grape [41], papaya [42], sorghum [43], cucumber [22], maize [44], soybean [45], Brachypodium [46], apple [47], castor bean [48], strawberry [49], and cacao [50]. Protein sequences of genes predicted from the fourteen plant genomes were downloaded from corresponding websites (Additional file 2). The 24,444 melon unigenes were then compared to these protein sequence databases using the NCBI BLAST (blastx) program. The complete comparative analysis results are shown in Additional file 3. At e value < 1e-05, approximately 85% of melon unigenes matched to proteins of cucumber, 75.4% to 79.2% of melon unigenes matched proteins of other dicot plants (Arabidopsis, poplar, apple, strawberry, cacao, grape, papaya, soybean, and castor bean), while 70.6% to 72.5% of melon unigenes matched proteins of monocot plants (rice, maize, sorghum, and Brachypodium). At a very stringent e value cutoff (e value < 1e-100), approximately 30% of melon unigenes matched cucumber proteins, 10.8% to 13.6% matched proteins of other dicot plants, and 7.9% to 8.5% matched proteins of monocot plants (Additional file 3). These matches represented the highly conserved proteins between melon and other plant species.

**Figure 3:**
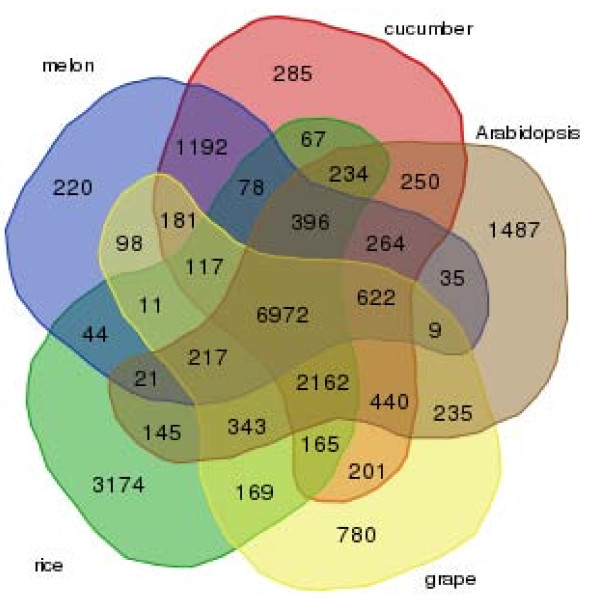
**Venn diagram of ortholog group distribution in melon, cucumber, Arabidopsis, grape, and rice**. Numbers in individual sections indicate the numbers of ortholog groups

We constructed families of homologous proteins using OrthoMCL [51] from protein sequences translated from melon unigenes with ESTScan [52] and from a wide phylogenetic range of representative plant organisms including cucumber, Arabidopsis, rice, and grape. These four organisms were chosen for the OrthoMCL analysis because cucumber, as melon, belongs to the *Cucurbitaceae *family; grape, cucumber and some cultivars of melon (e.g., Piel de sapo) are non-climacteric fleshy fruit; and Arabidopsis and rice represent the model systems for dicot and monocot plants, respectively. As shown in Figure 3, the analysis revealed 6,972 gene families that were distributed among the five genomes, which represented highly conserved gene families across dicot and monocot plant kingdoms. We also identified 181 gene families that were specific to fleshy fruit-bearing plants (melon, cucumber, and grape), 1,192 families specific to the *Cucurbitaceae *family (melon and cucumber), and 220 specific to melon. Functional analysis of melon unigenes using GO terms revealed that the 6,972 melon gene families common to the other four plant species were highly enriched with GO terms related to cellular process, metabolic process, and biosynthetic process (Additional file 4). This is consistent with a previous report [50]. Gene families specific to fleshy fruits were significantly enriched with GO terms related to hormone-mediated signaling pathway, response to biotic stimulus, and regulation of metabolic processes (Additional file 4); all these biological processes have been reported to be related to fleshy fruit development [53]. Gene families specific to the *Cucurbitaceae *family were significantly enriched with GO terms related to responses to various stimuli including responses to hormone and chemical stimuli (Additional file 4). Both melon and cucumber have diverse floral sex types and have long served as the primary model systems for sex determination studies [54]. It has been reported that a number of environment variables, such as light, temperature, water stress, and disease, as well as exogenous treatment with hormones or other growth-regulating substances, can directly influence floral sex determination [55,56]. Results obtained from the OrthoMCL analysis indicated that cucurbit specific gene families were enriched with such stimulus-responsive genes which might play roles in floral sex determination. Further studies, of course, are required to test this hypothesis. Finally, we found that gene families specific to melon mainly encompassed genes of unknown functions, which is consistent with findings reported in other plant species [50].

### Tissue-specific melon gene expression

**Figure 4:**
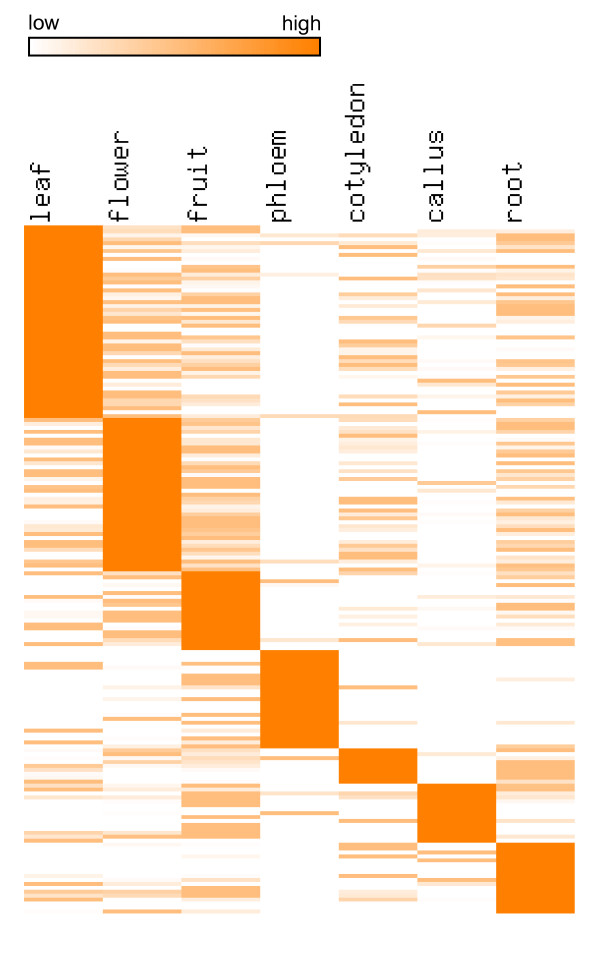
**Heatmap representation of expression profiles of melon tissue-specific genes**.

Melon cDNA libraries generated in the present study, as well as melon phloem EST libraries described in Omid et al. [29], were neither normalized nor subtracted; thus for these libraries, EST copy numbers can be used as an approximate estimation of gene expression levels in the corresponding tissues. The non-normalized and non-subtracted melon cDNA libraries were prepared from the following seven tissues: leaf, flower, fruit, phloem, cotyledon, callus, and root. Statistical analysis identified a total of 175 tissue-specific genes, among which 49, 39, 20, 25, 9, 15, and 18 were leaf, flower, fruit, phloem, cotyledon, callus, and root-specific, respectively (Additional file 5). Heatmap representation of expression profiles of these tissue-specific genes is shown in Figure 4. In most cases, genes expressed in specific tissues had putative functions or were involved in pathways known to be consistent with said tissue, e.g., leaf-specific genes were highly enriched with genes involved in photosynthesis, phloem-specific genes were highly enriched with genes encoding phloem filament proteins and phloem lectins, and callus-specific genes were highly enriched with genes involved in glycolysis, glucose metabolic process, hexose metabolic process, monosaccharide metabolic process, carbohydrate catabolic process, and alcohol metabolic process (Additional file 5). It is worth pointing out that some tissue-specific genes identified in leaf, cotyledon and root might be due to the infection of MNSV-Mα5. Indeed, functional analysis indicated that leaf, cotyledon and root-specific genes were enriched with GO terms such as response to stimulus and defense response (Additional file 5).

It is worth noting that one of the fruit-specific genes encoded 1-aminocyclopropane-1-carboxylate oxidase (ACO), the final enzyme in the biosynthesis of ethylene which is a plant hormone that regulates ripening of climacteric fruits [57]. Further detailed digital expression analysis of this gene (MU46283) revealed that, as expected, the gene was predominantly expressed in fruits of melon cultivars Dulce and Vedrantais, both of which are climacteric fruits; while none or very few ACO transcripts were detected in fruits of the two non-climacteric cultivars, PI161375 and Piel de Sapo T-111. In addition, two genes (MU45060 and MU46015) encoding acyl carrier proteins (ACPs) were highly and exclusively expressed in fruit tissues. ACPs are essential components of the fatty acid synthase complex and may be required to maintain the production of fruit aroma volatiles [58].

Interestingly, we found that genes involved in nucleosome and chromatin assembly (e.g., histones) and translation process (e.g., ribosomal proteins) were highly enriched in the list of flower-specific genes (Additional file 5). However, the exact role of these flower-specific genes in melon flower development remains unclear and further studies are required to clarify their functions in flower development.

### Marker discovery from melon EST sequences

Molecular markers are valuable resources for constructing high-density genetic maps, facilitating crop breeding and identifying traits of interest. Early melon genetic maps mainly used markers of Restriction Fragment Length Polymorphism (RFLP), Amplified Fragment Length Polymorphism (AFLP), and Random Amplified Polymorphic DNA (RAPD). However these types of markers are not user friendly as they are either labor intensive to generate, harbor low rates of polymorphism in melon [59], or are not readily transferred to other genotypes and populations [60]. With the accumulation of sequence information in melon during the past several years, markers of simple sequence repeats (SSRs) and single nucleotide polymorphisms (SNPs) are becoming more widely used in construction of melon genetic maps. These markers have the following advantages: they are hypervariable, multiallelic, codominant, locus-specific, and evenly distributed throughout the genome [60], and for markers derived from ESTs, they are directly linked to expressed genes. The melon EST sequence information generated in this and other studies has served as a major resource to generate new molecular markers (mainly SSRs and SNPs). Several recently constructed melon high-density genetic maps have already utilized SSR and SNP markers derived from EST sequences generated in the present study [18,19].

We first screened melon unigenes for the presence of di-, tri-, tetra-, penta- and hexa-nucleotide SSR motifs. We retrieved 4,068 SSR motifs in 3,279 melon unigenes. The major types of melon SSR motifs were tri-nucleotide, followed by di-nucleotide, tetra-nucleotide, penta-nucleotide and hexa-nucleotide (Table 4). The most frequent SSR motif was AAG/CTT (1,269; 31.2%), followed by AG/CT (1,134; 27.9%), AT/AT (364; 8.9%) and AAT/ATT (120; 2.9%). CG/CG (3) was the least frequent SSR motif identified in melon unigenes; possibly due to the fact that CpG sequences are normally highly methylated, which may further inhibit transcription [61]. These statistics are in agreement with previous reports of other plant species [26,62]. Primer pairs were designed for SSR motifs that had sufficient flanking sequences. The complete list of SSR motifs and their corresponding primer pair information is provided in Additional file 6.

**Table 4:** Statistics of melon simple sequence repeats (SSRs)

Unit size	Number of SSRs
di-nucleotide	1657
tri-nucleotide	2157
tetra-nucleotide	124
penta-nucleotide	51
hexa-nucleotide	79

ESTs generated in this (Table 1) and other studies [24,28,29] were from a diversity of melon cultivars. We expected that SNPs would be enriched in the melon EST dataset. Using very stringent criteria (see Methods for details), we identified a total of 3,073 high-quality SNPs in 1,331 unigenes, among which 1,972 were transitions, 976 were transversions, and 125 were single-base insertions or deletions (Table 5). The most frequent SNPs were C to T transitions (1,108; 36.1%), followed by A to G transitions (864; 28.1%) (Table 5). The complete list of SNPs identified from melon ESTs is provided in Additional file 7. Detailed information including alignments of sequences containing each individual SNP is also available at the Cucurbit Genomics Database [28]. Both SSRs and SNPs identified in the present study represent an important resource for genetic linkage mapping and marker-assisted breeding in melon and closely related crops. As stated above, they have already been used for these purposes.

**Table 5:** Statistics of melon single nucleotide polymorphisms (SNPs)

SNP	No. SNPs	type	total
A -> G	864	Transition	1972
C -> T	1108		

A -> C	255		
A -> T	289	transversion	976
C -> G	210		
G -> T	222		

T -> -	40		
A -> -	38		
G -> -	23	indel	125
C -> -	24		

## Conclusion

We present the analysis of more than 71,000 and 22,000 melon ESTs from eleven full-length enriched and four standard cDNA libraries, respectively. These libraries were constructed from a range of tissues and melon genotypes. Analysis of approximately 1,400 melon full-length transcripts identified from this EST collection indicated that melon transcripts had 5' and 3' UTRs of similar size as those of tomato, while longer than those of other dicot plants that we investigated. Comparative analysis between melon ESTs and other plant genomes allowed us to identify a number of highly conserved gene families across the plant kingdom, as well as gene families specific to fleshy-fruit bearing plants, to the *Cucurbitaceae *family, and to melon. Digital expression analysis identified genes showing significant tissue-specific expression and this resource remains to be further exploited from the perspective of mining expression data. Furthermore, SSR and SNP markers were also identified in this melon EST collection and recent research activities have begun to utilize these resources to construct high-density genetic maps [18,19]. Overall the availability of a large collection of melon ESTs from full-length enriched and standard cDNA libraries will not only facilitate the annotation of the melon genome, which is currently being sequenced by the Spanish Genomics Initiative, but also provide a valuable resource for further functional and comparative genomics analysis, and for future improvement of breeding programs of melon and closely related species.

## Methods

### Plant material

Fruits of the four genotypes were collected at four developmental stages: 10, 20, 30 Days After Anthesis (DAA) and at the mature stage. The mature stage was determined based on the formation of the abscission zone in the two climacteric genotypes Dulce and Vedrantais (42 and 32 DAA, respectively) and based on highest Total Soluble Solids (TSS) for the two non-climacteric fruits PI161375 and Piel de sapo (42 and 45 DAA, respectively). Hermaphrodite flowers were collected on secondary axes at three developmental stages, C1, C3, and C5, which correspond to initial, medium and late developmental stages of flowers before anthesis, respectively (Caño-Delgado, unpublished). Specifically, C1 is the most initial stage where the flowers are around 1 mm in the longitudinal axis, C3 is the stage where the future fruit shape is already defined and first stamens are visible, and C5 is the stage just before anthesis (1-2 cm). MNSV-Mα5 infected cotyledons, leaves and roots were produced from melon cultivar Piel de Sapo T111 grown in growth chamber with a 16-hour, 25°C light and 8-hour, 18°C dark regime. Specifically, nine-day old cotyledons were inoculated mechanically with fresh inoculums of MNSV-Mα5 and harvested after 4 days when necrotic lesions started to appear with high incidence. Leaves and roots were harvest 10 and 8-10 days after inoculation with MNSV-Mα5, respectively. Undifferentiated callus growth was induced from cotyledon sections of the four cultivars (Dulce, Piel de Sapo T111, PI161375, and Vedrantais). Fifty seeds from each genotype were surfaced-sterilized in 70% ethanol for 2 min, followed by 1% (w/v) NaOCl with 0.1% (v/v) Tween-20 for 20 min, and rinsed three times with sterile distilled water. Under a dissecting microscope, seed coats were removed, a small incision was done on the integuments, and embryos were hydrated overnight in sterile distilled water. Embryo axis was removed from the de-coated seeds. Depending on the genotype, four to six transversal cotyledon sections were dissected from each seed and cultured in Petri dishes containing callus induction medium. Cultures were incubated in the dark, at 28°C, and subcultured every three weeks to fresh medium. Callus induction medium was the MS (Murashige and Skoog), supplemented with 30g·L^-1 ^sucrose, 8g·L^-1 ^Bacto agar (Difco Laboratories, Detroit), 5uM 2,4-dichlorophenoxyacetic acid (2,4-D), and 1uM Kinetin (6-furfurylaminopurine). Five months after initiation, 100 Petri dishes, 10-cm-wide, with six to eight calli were produced from each genotype.

### Total RNA preparation, cDNA library construction and cDNA clone sequencing

Total RNAs from callus and MNSV-infected tissues were extracted following the TRI-reagent (SIGMA) protocol, including two additional chloroform purification steps. Fruit total RNAs were prepared from slices of the fruit that included both flesh and rind using the protocol described by Portnoy et al. [25]. Melon flower total RNA was extracted from hermaphrodite flowers using TRIzol reagent (Invitrogen) and chlorophorm, following the protocol described by Cuperus et al. [63].

All RNA samples were submitted to one extra cleaning step on RNeasy columns (Qiagen) and purified on a poly(A) track system (Promega). For cDNA library construction, fruit and flower RNAs were pooled, respectively, by mixing equal amount of RNA from each developmental stage. Full-length enriched cDNA libraries were constructed with the RNA Captor protocol, as described previously [64], and the four standard callus cDNA libraries were constructed using the pBluescript II XR cDNA Library Construction Kit (Stratagene) according to the manufacturer's instructions. A subset of clones was randomly selected from each cDNA library. Clones from full-length enriched cDNA libraries were sequenced at Genoscope (Evry, France) and those from standard cDNA libraries at Arizona Genome Institute.

### EST sequence processing, assembly, and annotation

The raw chromatogram files were base-called with phred [65]. Vector, adaptor and low-quality bases (a 20-bp window with an average error rate > 0.01) were trimmed from the raw EST sequences using LUCY [66]. The resulting sequences were then screened against the NCBI UniVec database, *E. coli *genome, and melon ribosomal RNA sequences using SeqClean [67], to remove possible contaminations of these sequences. Sequences shorter than 100 bp were discarded. The resulting high quality melon ESTs have been deposited in GenBank dbEST database under accession numbers JG463773-JG557528 and are also available at the Cucurbit Genomics Database [28].

Melon ESTs were assembled into unigenes using iAssembler [68] with minimum overlap of 40 bp and minimum percent identity of 97. Melon unigene sequences were compared against GenBank non-redundant (nr) and UniProt [69] protein databases using the NCBI BLAST program with a cutoff e value of 1e-5. The unigene sequences were translated into proteins using ESTScan [52] and the translated proteins were then compared to pfam domain database [30] using HMMER3 [70]. Gene Ontology (GO) terms and plant-specific GO slim ontology [31] were assigned to each unigene based on terms annotated to its corresponding homologues in the UniProt database and domains in pfam database. Melon biochemical pathways were predicted from the unigenes using the Pathway Tools program [32] and a melon biochemical pathway database was constructed and is available at the Cucurbit Genomics Database [28].

### Full-length transcript identification and analysis

Unigenes containing both 5' and 3' sequences of at least one clone from the full-length enriched cDNA libraries were identified as full-length transcripts. The complete CDS were identified using the *getorf *application in the EMBOSS package [71]. CDS were also identified based on the ESTScan translations and CDS identified from the two approaches were integrated. 5' and 3' UTRs were then extracted from each candidate full-length transcript. Codon usages were calculated with the *cusp *program in the EMBOSS package [71].

### Comparative genomics analysis

Melon unigenes were compared to protein databases of fourteen plant species whose genomes have been fully sequenced (Additional file 2) using the NCBI BLAST program with an e value cutoff of 1e-5. Furthermore, ortholog groups of protein sequences for melon (ESTScan translated proteins), Arabidopsis, rice, cucumber, and grape were identified using the orthoMCL program, which performs an all-against-all BLAST comparison of protein sequences with subsequent Tribe-Markov clustering [51. Venn diagram showing the distribution of shared gene families among melon, Arabidopsis, rice, cucumber and grape was created with Venn Diagrams [72]. Enriched GO terms of melon unigenes in each list of specific ortholog groups were identified using GO::TermFinder [73] with corrected p values (False Discovery Rate (FDR); [74]) less than 0.05.

### Identification of tissue-specific genes

All normalized or subtracted cDNA libraries (e.g., libraries described in Gonzalez-Ibeas et al [24]) were excluded in the digital expression analysis. Pair-wise comparisons between fruit, flower, callus, leaf, root, cotyledon (Table 1), and phloem [29] were performed with the R statistic described in Stekel et al. [75] to identify differentially expressed genes. Only genes with a total of at least five EST members in the two compared tissues were included in the analysis. Raw p values from the R statistic were corrected for multiple testing using the FDR [74]. Tissue-specific genes were identified if the genes were significantly up-regulated (ratio > 2 and FDR < 0.05) in the tissue when compared to all other tissues. Enriched GO terms in each list of tissue-specific genes were identified using GO::TermFinder [73], requiring p values adjusted for multiple testing (FDR) to be less than 0.05.

### Identification of SSRs and SNPs

SSRs in melon unigene sequences were identified using the MISA program [76]. The minimum repeat number was six for dinucleotide and five for tri-, tetra-, penta- and hexa-nucleotide. Primer pairs flanking each SSR loci were designed using the Primer3 program [77].

SNPs in the cDNA sequences between different melon cultivars were identified with PolyBayes [78], which takes into account both the depth of the coverage and quality of the bases. To further eliminate errors introduced by PCR amplification during the cDNA synthesis step and to distinguish true SNPs from allele differences, we filtered PolyBayes results and only kept SNPs meeting both of the following two criteria: 1) at least 2X coverage at the potential SNP site for each cultivar; 2) no same bases at the potential SNP site between the two compared cultivars. The detailed information of all melon SSRs and SNPs is freely available at the Cucurbit Genomics Database [28].

## Authors' contributions

ZF, CC, TJ, JGM, ABe, JJG and MAA conceived and designed the study. JGM coordinated the ICuGI project. VT, RDS, VP, AMC, AICD and NK collected tissues and prepared RNA samples. CC, TJ, DJ, ABo and MEH constructed cDNA libraries. YZ, MH and ZF performed data analysis. ZF and CC wrote the manuscript. All authors approved the final manuscript.

## Supplementary Material

Additional file 1 **Codon usages of melon and Arabidopsis coding sequences**. The table provides the statistics of codon usages of melon and Arabidopsis coding sequences. Click here for file

Additional file 2 **Plant protein databases used for comparative genomics analysis**. The table provides the list of protein databases of plants with fully sequenced genomes that were used in the comparative analysis of melon unigenes. Click here for file

Additional file 3 **Comparative analysis of melon unigenes**. The table provides the statistics of comparison between melon unigenes and fourteen plant protein databases using the BLAST program. Click here for file

Additional file 4 **Enriched Gene Ontology (GO) terms in gene families**. The table provides the list of enriched GO terms identified from melon unigenes in gene families specific to the *Cucurbitaceae *family, to the fleshy fruit-bearing plant species, and common across the five plant species, respectively. Click here for file

Additional file 5 **Tissue-specific genes**. The file provides the list of genes that have tissue-specific expression and the list of GO terms enriched in each list of tissue-specific genes. Click here for file

Additional file 6 **Melon SSRs**. The table provides the list of SSRs identified from melon ESTs, their motif sequences and surrounding primer pair information. Click here for file

Additional file 7 **melon SNPs**. The table provides the list of SNPs identified from melon ESTs. Click here for file

## References

[B1] Jeffrey C A new system of *Cucurbitaceae* Bot Zhurn 2005 90 332 335

[B2] Jeffrey C De Wilde WJJO A review of the subtribe Thladianthinae (*Cucurbitaceae*) Bot Zhurn 2006 91 766 776

[B3] Boualem A Fergany M Fernandez R Troadec C Martin A A conserved mutation in an ethylene biosynthesis enzyme leads to andromonoecy in melons Science 2008 321 836 838 10.1126/science.1159023 18687965

[B4] Martin A Troadec C Boualem A Rajab M Fernandez R A transposon-induced epigenetic change leads to sex determination in melon Nature 2009 461 1135 1138 10.1038/nature08498 19847267

[B5] Haritatos E Keller F Turgeon R Raffinose oligosaccharide concentrations measured in individual cell and tissue types in *Cucumis melo L*. Leaves: implication for phloem loading Planta 1996 198 614 622 10.1007/BF00262649 28321672

[B6] Gomez G Torres H Pallas V Identification of translocatable RNA-binding phloem proteins from melon, potential components of the long-distance RNA transport system Plant J 2005 41 107 116 15610353 10.1111/j.1365-313X.2004.02278.x

[B7] Nunez-Palenius HG Gomez-Lim M Ochoa-Alejo N Grumet R Lester G Cantliffe DJ Melon fruits: genetic diversity, physiology, and biotechnology features Crit Rev Biotechnol 2008 28 13 55 10.1080/07388550801891111 18322855

[B8] Giovannoni JJ Fruit ripening mutants yield insights into ripening control Curr Opin Plant Biol 2007 10 283 289 10.1016/j.pbi.2007.04.008 17442612

[B9] Gonda I Bar E Portnoy V Lev S Burger J Schaffer AA Tadmor Y Gepstein S Giovannoni JJ Katzir N Lewinsohn E Branched-chain and aromatic amino acid catabolism into aroma volatiles in Cucumis melo L. fruit J Exp Bot 2010 61 1111 1123 10.1093/jxb/erp390 20065117 PMC2826658

[B10] Dai N Cohen S Portnoy V Tzuri G Harel-Beja R Pompan-Lotan M Carmi N Zhang G Diber A Pollock S Metabolism of soluble sugars in developing melon fruit: a global transcriptional view of the metabolic transition to sucrose accumulation Plant Mol Biol 2011 in press 10.1007/s11103-011-9757-1 21387125

[B11] Arumuganathan K Earle ED Nuclear DNA content of some important plant species Plant Molecular Biology Reporter 1991 9 208 218 10.1007/BF02672069

[B12] van Leeuwen H Monfort A Zhang HB Puigdomenech P Identification and characterization of a melon genomic region containing a resistance gene cluster from a constructed BAC library. Microcolinearity between *Cucumis melo *and *Arabidopsis thaliana* Plant Mol Biol 2003 51 703 718 10.1023/A:1022573230486 12678558

[B13] Morales M Orjeda G Nieto C van Leeuwen H Monfort A A physical map covering the *nsv *locus that confers resistance to Melon necrotic spot virus in melon (*Cucumis melo L.*) Theor Appl Genet 2005 111 914 922 10.1007/s00122-005-0019-y 16052354

[B14] Gonzalez VM Rodriguez-Moreno L Centeno E Benjak A Garcia-Mas J Puigdomenech P Aranda MA Genome-wide BAC-end sequencing of *Cucumis melo *using two BAC libraries BMC Genomics 2011 11 11 10.1186/1471-2164-11-618 PMC3091759 21054843

[B15] Gonzalez VM Garcia-Mas J Arus P Puigdomenech P Generation of a BAC-based physical map of the melon genome BMC Genomics 2010 11 339 10.1186/1471-2164-11-339 20509895 PMC2894041

[B16] Perin C Gomez-Jimenez M Hagen L Dogimont C Pech JC Molecular and genetic characterization of a non-climacteric phenotype in melon reveals two loci conferring altered ethylene response in fruit Plant Physiol 2002 129 300 309 10.1104/pp.010613 12011360 PMC155893

[B17] Fernandez-Silva I Eduardo I Blanca J Esteras C Pico B Bin mapping of genomic and EST-derived SSRs in melon (*Cucumis melo L.*) Theor Appl Genet 2008 118 139 150 10.1007/s00122-008-0883-3 18806992

[B18] Deleu W Esteras C Roig C Gonzalez-To M Fernandez-Silva I Gonzalez-Ibeas D Blanca J Aranda MA Arus P Nuez F Monforte AJ Pico MB Garcia-Mas J A set of EST-SNPs for map saturation and cultivar identification in melon BMC Plant Biol 2009 9 90 10.1186/1471-2229-9-90 19604363 PMC2722630

[B19] Harel-Beja R Tzuri G Portnoy V Lotan-Pompan M Lev S Cohen S Dai N Yeselson L Meir A Libhaber SE A genetic map of melon highly enriched with fruit quality QTLs and EST markers, including sugar and carotenoid metabolism genes Theor Appl Genet 2010 121 511 533 10.1007/s00122-010-1327-4 20401460

[B20] Mascarell-Creus A Canizares J Vilarrasa-Blasi J Mora-Garcia S Blanca J An oligo-based microarray offers novel transcriptomic approaches for the analysis of pathogen resistance and fruit quality traits in melon (*Cucumis melo L.*) BMC Genomics 2009 10 467 10.1186/1471-2164-10-467 19821986 PMC2767371

[B21] Dahmani-Mardas F Troadec C Boualem A Lévêque S Alsadon AA Engineering melon plants with improved fruit shelf life using the TILLING Approach PLoS ONE 2010 5 e15776 10.1371/journal.pone.0015776 21209891 PMC3012703

[B22] Huang S Li R Zhang Z Li L Gu X Fan W Lucas WJ Wang X Xie B Ni P The genome of the cucumber, *Cucumis sativus *L Nat Genet 2009 41 1275 1281 10.1038/ng.475 19881527

[B23] Rudd S Expressed sequence tags: alternative or complement to whole genome sequences? Trends Plant Sci 2003 8 321 329 10.1016/S1360-1385(03)00131-6 12878016

[B24] Gonzalez-Ibeas D Blanca J Roig C Gonzalez-To M Pico B Truniger V Gomez P Deleu W Cano-Delgado A Arus P MELOGEN: an EST database for melon functional genomics BMC Genomics 2007 8 306 10.1186/1471-2164-8-306 17767721 PMC2034596

[B25] Portnoy V Diber A Pollock S Karchi H Lev S Tzuri G Harel-Beja R Forer R Portnoy VH Lewinsohn E Tadmor Y Burger J Schaffer A Katzir N Use of non-normalized, non-amplified cDNA for 454-based RNA-seq of fleshy melon fruit The Plant Genome 2011

[B26] Guo S Zheng Y Joung JG Liu S Zhang Z Crasta OR Sobral BW Xu Y Huang S Fei Z Transcriptome sequencing and comparative analysis of cucumber flowers with different sex types BMC Genomics 2010 11 384 10.1186/1471-2164-11-384 20565788 PMC2897810

[B27] Blanca J Cañizares J Roig C Ziarsolo P Nuez F Picó B Transcriptome characterization and high throughput SSRs and SNPs discovery in *Cucurbita pepo *(Cucurbitaceae) BMC Genomics 2011 12 104 10.1186/1471-2164-12-104 21310031 PMC3049757

[B28] Cucurbit Genomics Database http://www.icugi.org

[B29] Omid A Keilin T Glass A Leshkowitz D Wolf S Characterization of phloem-sap transcription profile in melon plants J Exp Bot 2007 58 3645 3656 10.1093/jxb/erm214 17928373

[B30] Finn RD Mistry J Tate J Coggill P Heger A Pollington JE Gavin OL Gunasekaran P Ceric G Forslund K The Pfam protein families database Nucleic Acids Res 2010 38 D211 222 10.1093/nar/gkp985 19920124 PMC2808889

[B31] Plant specific GO slims http://www.geneontology.org/GO.slims.shtml

[B32] Karp PD Paley S Romero P The Pathway Tools software Bioinformatics 2002 18 S225 S232 10.1093/bioinformatics/18.suppl_1.S225 12169551

[B33] Aoki K Yano K Suzuki A Kawamura S Sakurai N Suda K Kurabayashi A Suzuki T Tsugane T Watanabe M Large-scale analysis of full-length cDNAs from the tomato (*Solanum lycopersicum*) cultivar Micro-Tom, a reference system for the Solanaceae genomics BMC Genomics 2010 11 210 10.1186/1471-2164-11-210 20350329 PMC2859864

[B34] Yamada K Lim J Dale JM Chen H Shinn P Palm CJ Southwick AM Wu HC Kim C Nguyen M Empirical analysis of transcriptional activity in the Arabidopsis genome Science 2003 302 842 846 10.1126/science.1088305 14593172

[B35] Umezawa T Sakurai T Totoki Y Toyoda A Seki M Ishiwata A Akiyama K Kurotani A Yoshida T Mochida K Sequencing and analysis of approximately 40,000 soybean cDNA clones from a full-length-enriched cDNA library DNA Res 2008 15 333 346 10.1093/dnares/dsn024 18927222 PMC2608845

[B36] Ralph SG Chun HJ Cooper D Kirkpatrick R Kolosova N Gunter L Tuskan GA Douglas CJ Holt RA Jones SJ Analysis of 4,664 high-quality sequence-finished poplar full-length cDNA clones and their utility for the discovery of genes responding to insect feeding BMC Genomics 2008 9 57 10.1186/1471-2164-9-57 18230180 PMC2270264

[B37] Jia J Fu J Zheng J Zhou X Huai J Wang J Wang M Zhang Y Chen X Zhang J Zhao J Su Z Lv Y Wang G Annotation and expression profile analysis of 2073 full-length cDNAs from stress-induced maize (*Zea mays *L.) seedlings Plant J 2006 48 710 727 10.1111/j.1365-313X.2006.02905.x 17076806

[B38] Arabidopsis Genome Initiative Analysis of the genome sequence of the flowering plant *Arabidopsis thaliana* Nature 2000 408 796 815 10.1038/35048692 11130711

[B39] International Rice Genome Sequencing Project The map-based sequence of the rice genome Nature 2005 436 793 800 10.1038/nature03895 16100779

[B40] Tuskan GA Difazio S Jansson S Bohlmann J Grigoriev I Hellsten U Putnam N Ralph S Rombauts S Salamov A The genome of black cottonwood, *Populus trichocarpa *(Torr. & Gray) Science 2006 313 1596 1604 10.1126/science.1128691 16973872

[B41] Jaillon O Aury JM Noel B Policriti A Clepet C Casagrande A Choisne N Aubourg S Vitulo N Jubin C The grapevine genome sequence suggests ancestral hexaploidization in major angiosperm phyla Nature 2007 449 463 467 10.1038/nature06148 17721507

[B42] Ming R Hou S Feng Y Yu Q Dionne-Laporte A Saw JH Senin P Wang W Ly BV Lewis KL The draft genome of the transgenic tropical fruit tree papaya (*Carica papaya *Linnaeus) Nature 2008 452 991 996 10.1038/nature06856 18432245 PMC2836516

[B43] Paterson AH Bowers JE Bruggmann R Dubchak I Grimwood J Gundlach H Haberer G Hellsten U Mitros T Poliakov A The *Sorghum bicolor *genome and the diversification of grasses Nature 2009 457 551 556 10.1038/nature07723 19189423

[B44] Schnable PS Ware D Fulton RS Stein JC Wei F Pasternak S Liang C Zhang J Fulton L Graves TA The B73 maize genome: complexity, diversity, and dynamics Science 2009 326 1112 1115 10.1126/science.1178534 19965430

[B45] Schmutz J Cannon SB Schlueter J Ma J Mitros T Nelson W Hyten DL Song Q Thelen JJ Cheng J Genome sequence of the palaeopolyploid soybean Nature 2010 463 178 183 10.1038/nature08670 20075913

[B46] International Brachypodium Initiative Genome sequencing and analysis of the model grass *Brachypodium distachyon* Nature 2010 463 763 768 10.1038/nature08747 20148030

[B47] Velasco R Zharkikh A Affourtit J Dhingra A Cestaro A Kalyanaraman A Fontana P Bhatnagar SK Troggio M Pruss D The genome of the domesticated apple (*Malus x domestica *Borkh.) Nat Genet 2010 42 833 839 10.1038/ng.654 20802477

[B48] Chan AP Crabtree J Zhao Q Lorenzi H Orvis J Puiu D Melake-Berhan A Jones KM Redman J Chen G Draft genome sequence of the oilseed species Ricinus communis Nat Biotechnol 2010 28 951 956 10.1038/nbt.1674 20729833 PMC2945230

[B49] Shulaev V Sargent DJ Crowhurst RN Mockler TC Folkerts O Delcher AL Jaiswal P Mockaitis K Liston A Mane SP The genome of woodland strawberry (*Fragaria vesca*) Nat Genet 2011 43 109 116 10.1038/ng.740 21186353 PMC3326587

[B50] Argout X Salse J Aury JM Guiltinan MJ Droc G Gouzy J Allegre M Chaparro C Legavre T Maximova SN The genome of *Theobroma cacao* Nat Genet 2011 43 101 108 10.1038/ng.736 21186351

[B51] Li L Stoeckert CJJ Roos DS OrthoMCL: identification of ortholog groups for eukaryotic genomes Genome Res 2003 13 2178 2189 10.1101/gr.1224503 12952885 PMC403725

[B52] Iseli C Jongeneel CV Bucher P ESTScan: a program for detecting, evaluating, and reconstructing potential coding regions in EST sequences Proc Int Conf Intell Syst Mol Biol 1999 138 148 10786296

[B53] Giovannoni JJ Genetic regulation of fruit development and ripening Plant Cell 2004 16 S170 S180 10.1105/tpc.019158 15010516 PMC2643394

[B54] Tanurdzic M Banks JA Sex-determining mechanisms in land plants Plant Cell 2004 16 S61 S71 10.1105/tpc.016667 15084718 PMC2643385

[B55] Heslop-Harrison J The experimental modification of sex expression in flowering plants Biol Rev 1957 32 38 90 10.1111/j.1469-185X.1957.tb01576.x

[B56] Korpelainen H Labile sex expression in plants Biol Rev 1998 73 157 180 10.1017/S0006323197005148

[B57] Yang SF Hoffman NE Ethylene biosynthesis and its regulation in higher-plants Annu Rev Plant Physiol Plant Mol Biol 1984 35 155 189 10.1146/annurev.arplant.35.1.155

[B58] Schwab W Davidovich-Rikanati R Lewinsohn E Biosynthesis of plant-derived flavor compounds The Plant Journal 2008 54 712 732 10.1111/j.1365-313X.2008.03446.x 18476874

[B59] Shattuck-Eidens DM Bell RN Neuhausen SL Helentjaris T DNA sequence variation within maize and melon: observations from polymerase chain reaction amplification and direct sequencing Genetics 1990 126 207 217 1977657 10.1093/genetics/126.1.207 PMC1204125

[B60] Ezura H Fukino N Research tools for functional genomics in melon (Cucumis melo L.): Current status and prospects Plant Biotechnology 2009 26 359 368 10.5511/plantbiotechnology.26.359

[B61] Lister R O'Malley RC Tonti-Filippini J Gregory BD Berry CC Millar AH Ecker JR Highly integrated single-base resolution maps of the epigenome in Arabidopsis Cell 2008 133 523 536 10.1016/j.cell.2008.03.029 18423832 PMC2723732

[B62] Kantety RV La Rota M Matthews DE Sorrells ME Data mining for simple sequence repeats in expressed sequence tags from barley, maize, rice, sorghum and wheat Plant Mol Biol 2002 48 501 510 10.1023/A:1014875206165 11999831

[B63] Cuperus JT Montgomery TA Fahlgren N Burke RT Townsend T Sullivan CM Carrington JC Identification of MIR390a precursor processing-defective mutants in Arabidopsis by direct genome sequencing Proc Natl Acad Sci USA 2010 107 466 471 10.1073/pnas.0913203107 20018656 PMC2806713

[B64] Clepet C RNA Captor, a tool for RNA characterization PLoS ONE 2011 6 e18445 10.1371/journal.pone.0018445 21533245 PMC3076428

[B65] Ewing B Hillier L Wendl MC Green P Base-calling of automated sequencer traces using phred. I. Accuracy assessment Genome Res 1998 8 175 185 9521921 10.1101/gr.8.3.175

[B66] Chou HH Holmes MH DNA sequence quality trimming and vector removal Bioinformatics 2001 17 1093 1104 10.1093/bioinformatics/17.12.1093 11751217

[B67] SeqClean program http://compbio.dfci.harvard.edu/tgi/software

[B68] iAssembler program http://bioinfo.bti.cornell.edu/tool/iAssembler

[B69] Apweiler R Martin MJ O'Donovan C Magrane M Alam-Faruque Y Antunes R Barrell D Bely B Bingley M Binns D The Universal Protein Resource (UniProt) in 2010 Nucleic Acids Res 2010 38 D142 148 19843607 10.1093/nar/gkp846 PMC2808944

[B70] HMMER3 http://hmmer.janelia.org

[B71] Rice P Longden I Bleasby A EMBOSS: The European Molecular Biology Open Software Suite Trends in Genetics 2000 16 276 277 10.1016/S0168-9525(00)02024-2 10827456

[B72] Venn Diagrams http://bioinformatics.psb.ugent.be/webtools/Venn

[B73] Boyle EI Weng S Gollub J Jin H Botstein D Cherry JM Sherlock G GO:TermFinder: open source software for accessing Gene Ontology information and finding significantly enriched Gene Ontology terms associated with a list of genes Bioinformatics 2004 20 3710 3715 10.1093/bioinformatics/bth456 15297299 PMC3037731

[B74] Benjamini Y Hochberg Y Controlling the false discovery rate: a practical and powerful approach to multiple testing J R Stat Soc Ser B 1995 57 289 300

[B75] Stekel DJ Git Y Falciani F The comparison of gene expression from multiple cDNA libraries Genome Res 2000 10 2055 2061 10.1101/gr.GR-1325RR 11116099 PMC313085

[B76] MISA program http://pgrc.ipk-gatersleben.de/misa

[B77] Primer3 program http://frodo.wi.mit.edu

[B78] Marth GT Korf I Yandell MD Yeh RT Gu Z Zakeri H Stitziel NO Hillier L Kwok PY Gish WR A general approach to single-nucleotide polymorphism discovery Nat Genet 1999 23 452 456 10.1038/70570 10581034

