# Supplementary material for: Analysis of expressed sequence tags generated from full-length enriched cDNA libraries of melon
Source: BMC Genomics. 2011 May 20;12:252. doi: 10.1186/1471-2164-12-252 (PMC3118787; doi:10.1186/1471-2164-12-252)
Supplement: Additional file 1 — Codon usages of melon and Arabidopsis coding sequences. The table provides the statistics of codon usages of melon and Arabidopsis coding sequences. [file 1471-2164-12-252-S1.PDF]

Additional file 1: Codon usages of melon and Arabidopsis coding sequences

Codon	Amino acid	Fraction of codon usage[a]		Percentage of codon usage[b]	
		Melon	Arabidopsis	Melon	Arabidopsis
GCA	A	0.271	0.281	2.061	1.785
GCC	A	0.216	0.152	1.646	0.967
GCG	A	0.098	0.132	0.742	0.839
GCT	A	0.415	0.435	3.161	2.761
TGC	C	0.466	0.402	0.748	0.733
TGT	C	0.534	0.598	0.858	1.089
GAC	D	0.327	0.31	1.747	1.678
GAT	D	0.673	0.69	3.602	3.729
GAA	E	0.532	0.525	3.362	3.544
GAG	E	0.468	0.475	2.960	3.207
TTC	F	0.464	0.471	2.013	1.990
TTT	F	0.536	0.529	2.323	2.234
GGA	G	0.346	0.368	2.490	2.356
GGC	G	0.19	0.139	1.372	0.888
GGG	G	0.173	0.159	1.245	1.017
GGT	G	0.291	0.335	2.097	2.145
CAC	H	0.386	0.373	0.826	0.845
CAT	H	0.614	0.627	1.315	1.422
ATA	I	0.203	0.252	1.067	1.327
ATC	I	0.302	0.336	1.591	1.770
ATT	I	0.495	0.413	2.606	2.176
AAA	K	0.449	0.493	2.877	3.133
AAG	K	0.551	0.507	3.533	3.224
CTA	L	0.091	0.108	0.827	1.019
CTC	L	0.166	0.162	1.507	1.537
CTG	L	0.118	0.108	1.076	1.019
CTT	L	0.266	0.258	2.421	2.445
TTA	L	0.113	0.139	1.031	1.321
TTG	L	0.245	0.225	2.232	2.135
ATG	M	1	1	2.318	2.452
AAC	N	0.425	0.467	1.778	2.041
AAT	N	0.575	0.533	2.406	2.328
CCA	P	0.326	0.338	1.709	1.628
CCC	P	0.174	0.108	0.910	0.519
CCG	P	0.137	0.167	0.717	0.803
CCT	P	0.363	0.388	1.899	1.869
CAA	Q	0.565	0.564	1.918	1.992
CAG	Q	0.435	0.436	1.475	1.540
AGA	R	0.303	0.358	1.539	1.932

AGG	R	0.22	0.204	1.118	1.099
CGA	R	0.136	0.118	0.694	0.638
CGC	R	0.094	0.069	0.480	0.373
CGG	R	0.087	0.09	0.441	0.486
CGT	R	0.16	0.161	0.813	0.871
AGC	S	0.123	0.126	1.024	1.149
AGT	S	0.143	0.161	1.191	1.468
TCA	S	0.186	0.21	1.544	1.905
TCC	S	0.182	0.121	1.508	1.099
TCG	S	0.097	0.1	0.803	0.910
TCT	S	0.269	0.282	2.233	2.562
ACA	T	0.275	0.317	1.359	1.608
ACC	T	0.243	0.194	1.198	0.982
ACG	T	0.125	0.145	0.617	0.735
ACT	T	0.357	0.344	1.761	1.743
GTA	V	0.141	0.155	0.962	1.032
GTC	V	0.191	0.184	1.299	1.221
GTG	V	0.255	0.255	1.739	1.696
GTT	V	0.412	0.406	2.806	2.701
TGG	W	1	1	1.158	1.235
TAC	Y	0.462	0.466	1.335	1.310
TAT	Y	0.538	0.534	1.555	1.501
TAA	*	0.372	0.36	0.134	0.088
TAG	*	0.179	0.204	0.064	0.050
TGA	*	0.449	0.436	0.162	0.106

[a]Fraction of codon usage indicates the fraction of codon usages for each individual amino acids

[b]Percentage of codon usage indicates the percentage of codon usages among all codons
