# Supplementary material for: Analysis of expressed sequence tags generated from full-length enriched cDNA libraries of melon
Source: BMC Genomics. 2011 May 20;12:252. doi: 10.1186/1471-2164-12-252 (PMC3118787; doi:10.1186/1471-2164-12-252)
Supplement: Additional file 2 — Plant protein databases used for comparative genomics analysis. The table provides the list of protein databases of plants with fully sequenced genomes that were used in the comparative analysis of melon unigenes. [file 1471-2164-12-252-S2.PDF]

Additional file 2: Plant protein databases used for comparative genomics analysis

species	annotation version	protein database link
cucumber	version 2.0	http://www.icugi.org/cgi-bin/ICuGI/genome/cucumber/download.cgi
Arabidopsis	TAIR version 10	ftp://ftp.arabidopsis.org/home/tair/Sequences/blast_datasets/TAIR10_blastsets/TAIR10_pep_20101214
poplar	phytozome v6.0	ftp://ftp.jgi-psf.org/pub/JGI_data/phytozome/v6.0/Ptrichocarpa/annotation/
apple	version 1.0	http://www.rosaceae.org/system/files/apple_genome/Malus_x_domestica.v1.0.consensus_peptide.fa.gz
strawberry	NA	https://strawberry.plantandfood.co.nz/gbrowse/navbar/strawberry/DownloadData/vescagenemodels2.faa
cacao	version 1	http://cocoagendb.cirad.fr/gbrowse/download/Theobroma_cacao_v1.pep.faa.gz
grape	NA	http://www.genoscope.cns.fr/externe/Download/Projets/Projet_ML/data/12X/annotation/Vitis_vinifera_peptide.fa.gz
papaya	phytozome v6.0	ftp://ftp.jgi-psf.org/pub/JGI_data/phytozome/v6.0/Cpapaya/annotation/Cpapaya_113_peptide.fa.gz
soybean	phytozome v6.0	ftp://ftp.jgi-psf.org/pub/JGI_data/phytozome/v6.0/Gmax/annotation/Gmax_109_peptide.fa.gz
castor bean	release 0.1	ftp://ftp.tigr.org/pub/data/castorbean/release_0.1/TIGR_castorWGS_release_0.1.aa.fsa.gz
Brachypodium	version 1.0	http://files.brachypodium.org/Annotation/Bradi_1.0.pep.fa.gz
maize	release-4a.53	http://ftp.maizesequence.org/release-4a.53/filtered-set/ZmB73_4a.53_filtered_translations.fasta.gz
rice	version 6.1	ftp://ftp.plantbiology.msu.edu/pub/data/Eukaryotic_Projects/o_sativa/annotation_dbs/pseudomolecules/version_6.1/all.dir/all.pep
aorghum	phytozome v6.0	ftp://ftp.jgi-psf.org/pub/JGI_data/phytozome/v6.0/Sbicolor/annotation/Sbicolor_79_peptide.fa.gz
