# Supplementary material for: Analysis of expressed sequence tags generated from full-length enriched cDNA libraries of melon
Source: BMC Genomics. 2011 May 20;12:252. doi: 10.1186/1471-2164-12-252 (PMC3118787; doi:10.1186/1471-2164-12-252)
Supplement: Additional file 3 — Comparative analysis of melon unigenes. The table provides the statistics of comparison between melon unigenes and fourteen plant protein databases using the BLAST program. [file 1471-2164-12-252-S3.PDF]

Additional file 3: Comparative analysis of melon unigenes

Species	BLAST e value cutoff				
	**1e-5**	**1e-15**	**1e-25**	**1e-50**	**1e-100**
**cucumber**	20744 (84.9%)	19327 (79.1%)	17941 (73.4%)	14395 (58.9%)	7316 (29.9%)
**Arabidopsis**	18653 (76.3%)	16312 (66.7%)	14089 (57.6%)	9081 (37.2%)	2631 (10.8%)
**poplar**	19213 (78.6%)	17147 (70.1%)	15050 (61.6%)	10137 (41.5%)	3206 (13.1%)
**apple**	18889 (77.3%)	16722 (68.4%)	14587 (59.7%)	9615 (39.3%)	2902 (11.9%)
**strawberry**	19105 (78.2%)	16913 (69.2%)	14745 (60.3%)	9737 (39.8%)	2914 (11.9%)
**cacao**	19360 (79.2%)	17343 (70.9%)	15304 (62.6%)	10328 (42.3%)	3321 (13.6%)
**grape**	18674 (76.4%)	16538 (67.7%)	14448 (59.1%)	9532 (39%)	2815 (11.5%)
**papaya**	18431 (75.4%)	16211 (66.3%)	14016 (57.3%)	9028 (36.9%)	2634 (10.8%)
**soybean**	19083 (78.1%)	16950 (69.3%)	14837 (60.7%)	9870 (40.4%)	3096 (12.7%)
**castor bean**	18985 (77.7%)	16909 (69.2%)	14822 (60.6%)	9935 (40.6%)	3097 (12.7%)
**Brachypodium**	17669 (72.3%)	15037 (61.5%)	12681 (51.9%)	7580 (31%)	2059 (8.4%)
**maize**	17256 (70.6%)	14692 (60.1%)	12328 (50.4%)	7270 (29.7%)	1924 (7.9%)
**rice**	17687 (72.4%)	15090 (61.7%)	12724 (52.1%)	7658 (31.3%)	2070 (8.5%)
**sorghum**	17719 (72.5%)	15094 (61.7%)	12692 (51.9%)	7629 (31.2%)	2062 (8.4%)
